# Tinnitus alters resting state functional connectivity (RSFC) in human auditory and non-auditory brain regions as measured by functional near-infrared spectroscopy (fNIRS)

**DOI:** 10.1371/journal.pone.0179150

**Published:** 2017-06-12

**Authors:** Juan San Juan, Xiao-Su Hu, Mohamad Issa, Silvia Bisconti, Ioulia Kovelman, Paul Kileny, Gregory Basura

**Affiliations:** 1 Department of Otolaryngology/Head and Neck Surgery, Kresge Hearing Research Inst., The University of Michigan, 1100 W Medical Center Drive, Ann Arbor, MI, United States of America; 2 Center for Human Growth and Development, The University of Michigan, Ann Arbor, MI, United States of America; Harvard Medical School, UNITED STATES

## Abstract

Tinnitus, or phantom sound perception, leads to increased spontaneous neural firing rates and enhanced synchrony in central auditory circuits in animal models. These putative physiologic correlates of tinnitus to date have not been well translated in the brain of the human tinnitus sufferer. Using functional near-infrared spectroscopy (fNIRS) we recently showed that tinnitus in humans leads to maintained hemodynamic activity in auditory and adjacent, non-auditory cortices. Here we used fNIRS technology to investigate changes in resting state functional connectivity between human auditory and non-auditory brain regions in normal-hearing, bilateral subjective tinnitus and controls before and after auditory stimulation. Hemodynamic activity was monitored over the region of interest (primary auditory cortex) and non-region of interest (adjacent non-auditory cortices) and functional brain connectivity was measured during a 60-second baseline/period of silence before and after a passive auditory challenge consisting of alternating pure tones (750 and 8000Hz), broadband noise and silence. Functional connectivity was measured between all channel-pairs. Prior to stimulation, connectivity of the region of interest to the temporal and fronto-temporal region was decreased in tinnitus participants compared to controls. Overall, connectivity in tinnitus was differentially altered as compared to controls following sound stimulation. Enhanced connectivity was seen in both auditory and non-auditory regions in the tinnitus brain, while controls showed a decrease in connectivity following sound stimulation. In tinnitus, the strength of connectivity was increased between auditory cortex and fronto-temporal, fronto-parietal, temporal, occipito-temporal and occipital cortices. Together these data suggest that central auditory and non-auditory brain regions are modified in tinnitus and that resting functional connectivity measured by fNIRS technology may contribute to conscious phantom sound perception and potentially serve as an objective measure of central neural pathology.

## Introduction

Tinnitus, the phantom perception of sound, is highly prevalent with an estimated 10–15% of adults affected in the United States [1]. The underlying etiology of tinnitus is not well defined, yet is largely associated with peripheral ear pathology leading to aberrant neural activity within central auditory circuits [2, 3]. Auditory cortex in animal models of tinnitus is one region that shows increased spontaneous neural firing rates and enhanced neural synchrony [4]. These putative physiologic correlates of tinnitus in animals have yet to be explored or translated in humans and it is not known whether comparable objective indicators exist and are measurable. Knowledge gaps are mainly due to limited technology available to measure human central auditory circuits in real-time. Extraneous noise from the machine can limit fMRI imaging as it may confound recording environments in tinnitus, while EEG has relatively low spatial resolution. The ability to identify and measure putative correlates of tinnitus in humans is vital to understanding aberrant brain regions and circuits that could objectify the disease, and thereby, direct and monitor efficacy of targeted therapies.

Functional near-infrared spectroscopy (fNIRS) is a valuable imaging modality to investigate tinnitus in humans. It is non-invasive, portable, relatively inexpensive and virtually silent, thereby reducing potential confounding effects of extraneous noise during data collection [5]. It uses IR-light to measure oxygenated (HbO) and deoxygenated (HbR) hemoglobin to derive hemodynamic activity, and changes from baseline in this measured activity serve as a surrogate of central neural activity. Due to neurovascular coupling, changes in neural activity lead to measurable gradations of optical hemodynamic properties of brain tissue [6]. Simply put, when a specific brain region is activated, fNIRS measures changes in localized hemoglobin level as an index of neural response. The derived hemoglobin index has relatively higher temporal resolution and is a better direct metabolic marker than the widely used BOLD effect in fMRI that derives information only from the properties of HbR [7]. fNIRS is mainly limited by a short depth of penetration and low spatial resolution, both of which are in the order of centimeters [8]. Although this constrains the use of fNIRS to investigating superficial cortex, given its advantages over other imaging modalities, it is well suited to serve as a complimentary technology in neuroscience research.

The use of fNIRS in the study of tinnitus was first demonstrated by Schecklmann et al [9]. They found increased activation in the right auditory cortex of tinnitus participants that was thought to represent at least one aspect of the tinnitus percept. We recently demonstrated increased hemodynamic activity in auditory and select adjacent non-auditory cortices using fNIRS in human participants with tinnitus, while non-tinnitus controls showed deactivation in the corresponding region [10]. Increased baseline neural activity following auditory stimulation in that study implicates a potential objective tinnitus correlate in humans equivalent to increased spontaneous firing rates seen in animal models. Interestingly, these responses in humans were also found in adjacent non-auditory cortical regions sub-served by other sensory and motor systems suggesting that tinnitus percepts may originate outside of primary auditory regions that may ultimately influence sound perception. As such, we currently hypothesized that another potential objective correlate of tinnitus in humans may, in part, be the result of aberrant connectivity between auditory and non-auditory brain regions.

Resting state functional connectivity (RSFC) is the association of baseline activity between two brain regions [11]. Although functional connectivity portends anatomical/structural interactions, these are not interchangeable [12]. Additionally, functional connectivity does not assess activity at the individual neuron level and so it is distinct from neural synchrony or congruent associated firing rates between single units; a touted tinnitus correlate in animal models. By assessing RSFC, we obtain information regarding spatiotemporal patterns of hemodynamic responses across brain regions, which are thought to reflect plastic changes that play a role in both adaptive and maladaptive conditions [12, 13]. RSFC has been proposed to represent contextual influences of connections involved in local processing, connections between regions that are likely to work together in the future, or serve to coordinate neural activity [14]. This is of particular interest in the study of humans with tinnitus as parallels to phantom pain perception have been used to propose that functional connectivity with frontal and parietal cortices is required for a phantom stimulus to become a conscious percept [15]. Furthermore, it has been proposed that aberrant processing in brain networks involving sensory cortices can give rise to phantom perceptions [15].

In this study, we used the same human participants from our recently published data that revealed increased/maintained cortical hemodynamic responses during silence after randomized auditory stimulation in tinnitus [10]. Here, we evaluated baseline RSFC within and between the same auditory and non-auditory cortices before and after an auditory stimulation paradigm. RSFC was measured between all channel-pairs during the 60-second pre- and 60-second post-auditory stimulation paradigm baseline period of silence. Prior to stimulation, we observed decreased auditory cortical connectivity in tinnitus as compared to control. Interestingly, connectivity of the auditory cortex in tinnitus increased following auditory stimulation, whereas connectivity in controls decreased. Moreover, strength of connectivity between auditory cortex and fronto-temporal, fronto-parietal, temporal, occipito-temporal, and occipital cortices increased in tinnitus following sound stimulation. Together these data suggest that human central auditory and non-auditory regions are modified in tinnitus as evidenced by measurable changes in brain connectivity using fNIRS technology. These changes using this non-invasive technology may also serve as objective neural correlates of tinnitus.

## Materials and methods

### Participants

The University of Michigan Institutional Review Board approved the study and participants were reimbursed for time. Written informed consent was obtained from each participant after an extensive explanation of the protocol using non-invasive fNIRS technology. The same participants (plus an additional control) from our recently published data showing maintained/increased hemodynamic activity in tinnitus as compared to controls were used in this study [10]. All tinnitus participants suffered from constant, non-pulsatile phantom sound that was perceived equally in both ears or in the “head,” and none endorsed hyperacusis (hypersensitivity to sound). Eight non-tinnitus controls (average age: 25.4±7.3 years, 5 men) and ten adults with subjective, bilateral tinnitus (average age: 48.7±16 years; 6 men) participated in the study. To exclude the effects of hearing loss, normal or near-normal hearing participants were selected using pre-determined audiometric criteria, speech reception thresholds (SRTs), and word recognition scores (WRS). SRT is the lowest intensity (measured in dB HL) at which 50% of a standardized list of phonetically-balanced two-syllable words are correctly identified and repeated [10]. WRS measures the percentage of one-syllable words presented at a supra-threshold, conversational level (40–50 dB HL above SRT) identified correctly. Audiograms for all participants confirmed average pure-tone thresholds of less than 30dB HL across the measured frequency range (including 8000Hz). Further exclusion criteria included prior otologic surgery, unilateral tinnitus, any conductive hearing loss, or other potential tinnitus etiologies (e.g. skull base tumors, retrocochlear lesions, high dose aspirin, etc.). There were no statistical differences between the groups regarding SRT or WDS and Pearson correlation analysis yielded no correlation between age, hearing thresholds, or audiogram findings.

### fNIRS imaging

**Fig 1:**
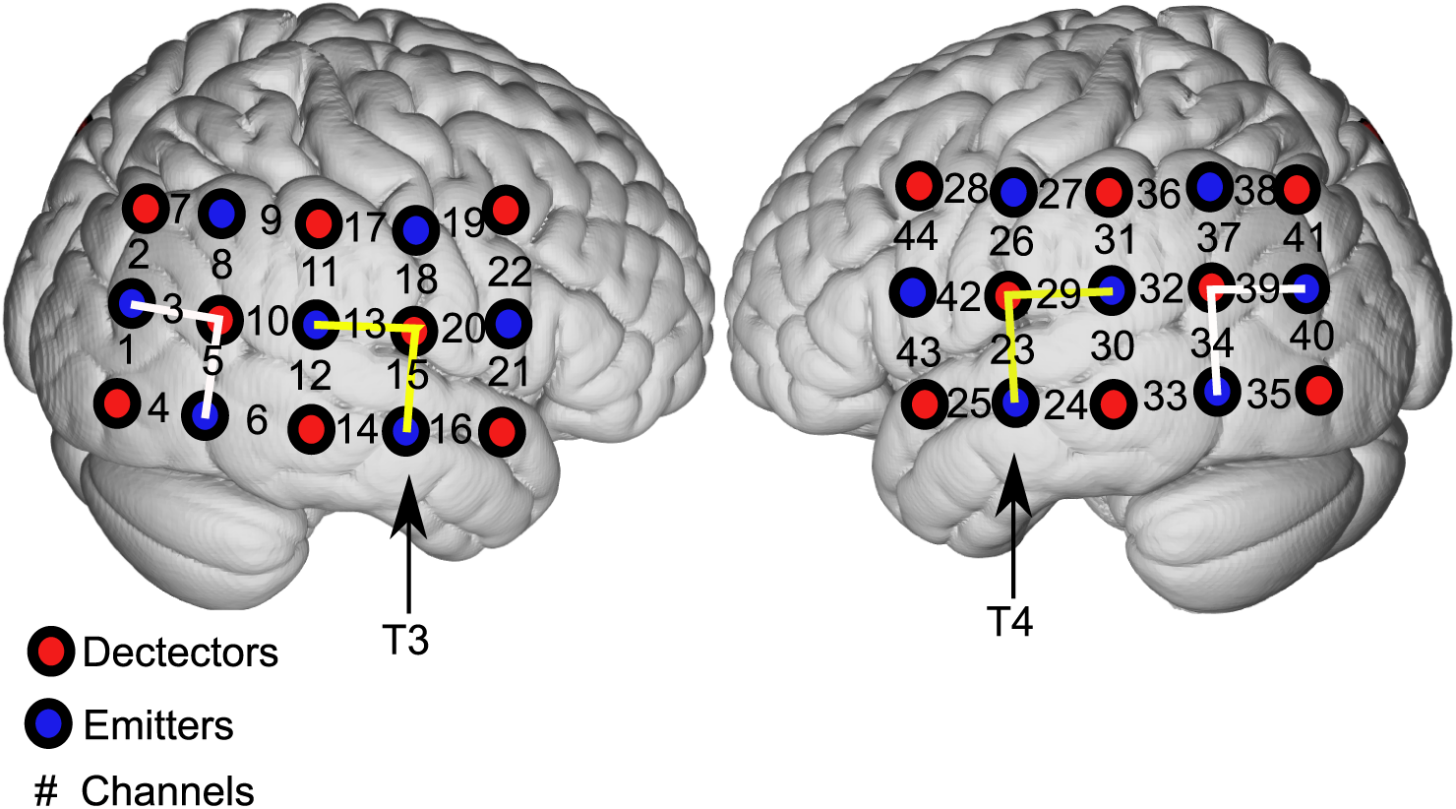
Channel configuration. Configuration of channels (numbers) with identified detectors (red circles) and emitters (blue circles) over the right and left cortical hemispheres. There are 8 detectors and 7 sources resulting in 22 channels per hemisphere. Interconnected blocks with the solid yellow line represent the region of interest (ROI; primary auditory regions; channels 13, 15, 23, and 29). Interconnected blocks with white line represent the *n-1* non-region of interest (non-ROI; (channels 3, 5, 34, and 39)). T3 and T4 are the reference points of the International 10–20 System [16].

This is the same recording environment used in previously published work [10]. We used a continuous wave fNIRS system (CW6, Techen, Inc., USA) with two wavelengths (690 and 830nm). We developed a cap configuration consisting of a silicone band containing 30 optodes (15 per hemisphere; Fig 1) that was wrapped circumferentially around the head and secured using Velcro straps. Each hemisphere contained 8 detectors and 7 emitters organized in 5 x 3 arrays over the frontal, temporal, parietal and occipital lobes. This source-detector pair arrangement yielded 22 channels on each hemisphere. The distance between each source and detector was 3 cm. To ensure consistent placement of the optodes throughout the experiment, the positions of T3 and T4 were confirmed prior to and following the recording sessions and pre- and post-experiment photographs were taken. Cap placement for all participants was deemed consistent. The data were collected at a sampling rate of 20Hz, except for one participant whose recording was performed at 50Hz and subsequently down-sampled.

### fNIRS anatomical localization/ROI selection

The region of interest (ROI) included primary auditory cortex (temporal lobe including superior temporal plane) and surrounding auditory belt regions (temporal and parietal cortices). Acknowledging the spatial resolution limits of fNIRS technology, both anatomical (10–20 EEG system, three-dimensional digitizer) and functional (normal brain response to auditory stimulation) strategies were utilized to identify ROI as published [10]. First, the International 10–20 System for EEG electrode placement [16] with bilateral T3/T4 coordinates for temporal lobe optode placements (Fig 1; [17, 18]) were utilized. Second, anatomical localization of ROI was achieved by isolating only those channels that in controls showed an increased average group hemodynamic activity response to auditory stimuli and subsequent declines during silence as reported [17, 19–23]. ROI (and “n-1” non-ROI; see below) was separately derived from two respective channels from each hemisphere (13 and 15: right hemisphere; 23 and 29: left hemisphere; Fig 1). Because responses were not statistically different between right and left hemispheres in all participants, ROI data from both hemispheres were pooled for analysis for all testing conditions.

The non-region of interest (non-ROI) is comprised of channels outside/adjacent to ROI minus an additional channel (“n−1”) as indicated by published criteria [10, 17]. These “n-1” non-ROI serve as a spatial control for analysis of ROI connectivity. In a separate analysis, all other channels were used as seeds to assess connectivity changes in areas other than auditory cortex. These channels are also referred to as non-ROI in the manuscript, but the reader should differentiate this generic use of the term from the specific situation involving “n-1” non-ROI. Channels corresponding to “n-1” non-ROI were 3 and 5 in the right hemisphere and 34 and 39 in the left hemisphere (Fig 1). Based on that selection criterion, these channels were identified as non-auditory (non-ROI) cortical regions that equated to Brodmann Areas 19 and 37. The additional groupings of channels representing frontal, temporal, parietal, occipital, fronto-temporal, fronto-parietal, temporo-parietal, occipito-parietal and occipito-temporal were based on the results of a three-dimensional (3D) digitizer (see below). Data from channels representing the same Brodmann Areas in separate hemispheres were pooled for the final analysis.

We used a 3D magnetic digitizer (Polhemus Patriot digitizer, Vermont, USA) to spatially locate fNIRS optode positions relative to ROI for analysis performed in this study by using five reference points (nasion, inion, right and left pre-auricular points, and midpoint of crown of the head, from International 10–20 system) [24]. The MATLAB-based software AtlasViewerGUI [25] was then used to transfer optode positions into Montreal Neurological Institute coordinates (a brain template composed of 152 adult MRIs) [26]. These coordinates were also used to generate the figures in the current manuscript. We then found the corresponding BA based on the automatic anatomical labeling (AAL) database and used that information to assign each channel to one of the groups as summarized in Table 1 [27].

**Table 1:** Corresponding Brodmann Areas and cortical regions for each channel.

Channel Number	Brodmann Areas	Anatomic location	Percent channels
1, 40	18, 19	Occipital	9.1%
4, 35	18, 19, 37	Occipital	
2, 41	19, 39	Occipito-parietal	4.6%
3, 39	7, 19	Occipito-temporal	9.1%
5, 34	19, 39	Occipito-temporal	
7, 38	7	Parietal	18.2%
9, 36	40	Parietal	
11, 31	40	Parietal	
17, 27	2, 40	Parietal	
8, 37	7, 19, 39, 40	Temporo-parietal	18.2%
10, 32	39, 40	Temporo-parietal	
13, 29	40, 42	Temporo-parietal	
15, 23	22, 40, 42	Temporo-parietal	
6. 33	21, 37	Temporal	18.2%
12, 30	22	Temporal	
14, 24	21, 22	Temporal	
16, 25	21, 22	Temporal	
18, 26	1, 2, 3, 4, 6, 40	Fronto-parietal	13.6%
19, 28	3, 4, 6	Fronto-parietal	
20, 42	1, 3, 4, 6, 43	Fronto-parietal	
21, 43	6, 22, 44	Fronto-temporal	4.6%
22, 44	6, 9	Frontal	4.6%

### Stimuli protocol

**Fig 2:**
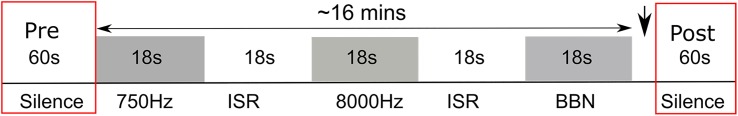
Study protocol. Schematic of block auditory testing paradigm. Participants passively listened to randomly selected pure tones (750 or 8000Hz) or broadband noise (BBN) for 18 seconds each, immediately followed or preceded by an inter-stimulus rest period (ISR) consisting of silence/absence of auditory stimulation for 18 seconds, for a total experiment run time of 18 minutes. A 60-second pre- and a separate 60-second post-stimulation paradigm baseline periods of silence were recorded from which the RSFC was derived. Black downward arrow depicts the four second wait period to allow activation levels to return to baseline.

Participants were exposed to a sound-field auditory block paradigm as published [10]. The auditory paradigm consisted of 54 alternating blocks of silence (inter-stimulus rest, ISR) and sound, each lasting 18 seconds. Sound blocks consisted of either pure tone (700Hz or 8000Hz) or broadband noise (BBN). They were selected to evaluate both partial and complete auditory cortical tonotopic activation as compared to ISR [28]. Each type of sound block was presented nine times in random order. Stimuli were generated using Audacity (GNU General Public License) and normalized with Praat 4.2 [29]. They were presented using E-prime (Psychology Software Tools, Inc., Pittsburgh, PA, USA) in sound-field orientation held at 70dB sound pressure level (SPL; Creative Inspire T12) by using two speakers located at approximately two feet from participants. Tinnitus and control participants recruited had no differences in hearing thresholds and this configuration achieved a consistent SPL that was above the hearing threshold for all participants, at similar sensation levels. To prevent motion artifact without formal head fixation or a rest platform, participants were instructed to visually fixate on a “plus sign” target displayed on a computer monitor located within arm’s length of where they were seated. They were instructed to stay awake, remain as still as possible, and simply listen throughout the 20-minute recording session. Our study focused on connectivity during the 60 seconds prior to the stimuli and the 60 seconds following it. The starting point of the post-stimulation time period was 4 seconds after the last stimulus to allow hemodynamic response to return to baseline (Fig 2) [30].

### Data analysis

All data were pre-processed using Homer2 software [30] based on MATLAB (Mathworks, MA, USA). Raw optical intensity data series (voltage) were initially converted into changes in optical intensity. The E-Prune channel function was used to exclude channels with very low signal to noise ratio optical intensity from the analyses [31]. The parameter α was set to 0.1 [31–33]. The data was band-pass filtered using Butterworth filters between 0.01Hz and 0.08Hz to eliminate cardiac and respiration induced hemodynamic fluctuations, remove signal noise from the instrument, and to obtain the low frequency spontaneous fluctuations necessary for RSFC calculation [34]. Optical density data were then converted into concentration changes using the modified Beer-Lambert law (MBLL) with a partial path length factor for both wavelengths of 6.0. This procedure was conducted in each condition and group for oxy-hemoglobin (HbO) and deoxy-hemoglobin (HbR), separately [32, 35]. The above methodology was used to process the entire 20-minute recording for each participant, which includes the stimulation period and the pre- and post-stimulation resting periods (see Fig 2). As a result, a common baseline was used for both the pre- and post-stimulation analysis. The connectivity was then determined only for the pre- and post-stimulation periods.

### Statistical analysis

**Fig 3:**
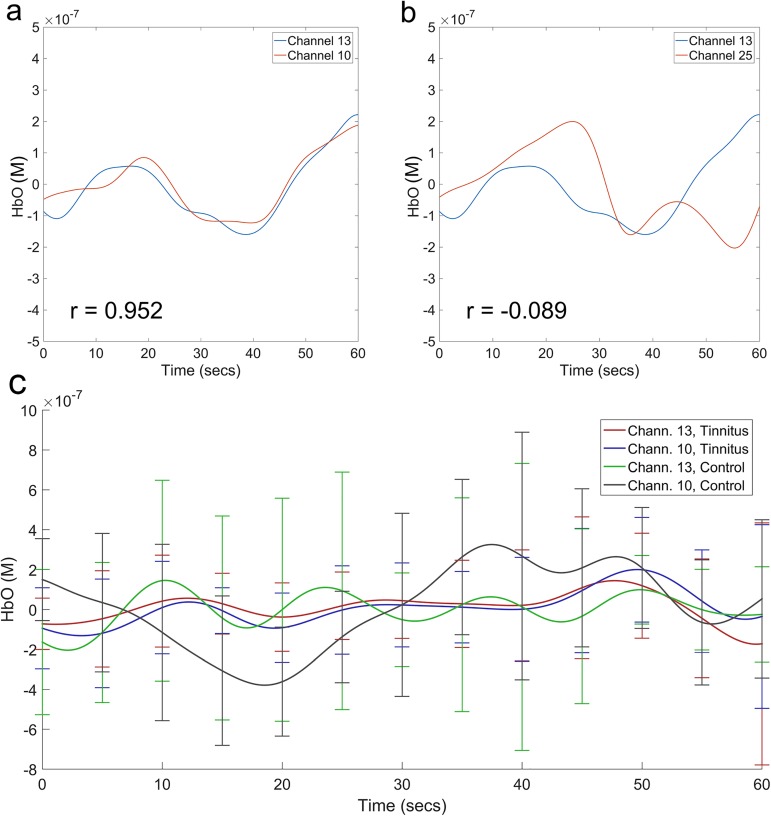
Demonstration of high and low correlated channels for participants during auditory pre-stimulation. The HbO concentration data (μM) of tinnitus participant number 10 in the pre-stimulation condition of (a) two highly correlated channels (channels 13 and 10; r = 0.952) and (b) two lowly correlated channels (channels 13 and 25; r = -0.089). (c) Mean hemodynamic response for channels 13 and 10 for the tinnitus and control groups with the standard deviation at select time points.

Pearson correlation coefficients (*r*) were obtained for each combination of channel-pairs for each participant [36]. This yielded 43 connection values for each channel per participant, which in turn yielded 40 connection values for the pooled ROI and pooled “n-1” non-ROI. Statistical analysis was focused on HbO since it constitutes a greater portion of signal from cortex (76%) compared to HbR (19%) [37] and the signal-to-noise contrast for HbO is better than HbR [38]. Linear regression of data from HbO and HbR for each of the four ROI seeds for each stimulation condition and patient group showed statistically significant correlations (lowest r = 0.4812, p-value <0.001). This suggests that both parameters are a result of a similar underlying physiologic process, and therefore, the parameter with the strongest signal is a more appropriate one to use for analysis [23], which is HbO. All channels were subsequently used for statistical analysis. A demonstration of high and low correlated channels is shown in Fig 3. To perform statistical analysis and to determine average values when pooling multiple channels, Fisher’s transformation was used (Eq 1). Fisher transformation is a stabilizing analysis that is necessary since the variance of Pearson correlation coefficients changes depending on proximity to 0 [39]. Statistical significance testing of the correlation coefficients used the null hypothesis that the mean of the sample equaled zero. The p-values were calculated using 2-tailed Student’s t-tests using standard deviation shown in Eq 2 [39]. Averages represent data that has been transformed back to a Pearson correlation coefficient using the inverse Fisher transformation (Eq 3) after the corresponding data analysis was performed [39]. To assess the validity of pooling data across hemispheres, linear regressions were used to compare the connectivity values of the ROI and “n-1” non-ROI channels on one hemisphere to those of the corresponding channels on the contralateral hemisphere. We checked this for tinnitus and control groups during the pre- and post-stimulation periods and found no asymmetry in any comparison (all p < 0.0001). All data analysis was performed using built-in MATLAB functions whenever possible, and originally developed MATLAB scripts when not.

$$z=.5\times ln\;\left(\frac{1+r}{1-r}\right)=arctanh(r)$$ (1)

$$\sigma_{z}=\frac{1}{\sqrt{N-3}}$$ (2)

$$r=\frac{e^{2z}-1}{e^{2z}+1}=tanh(z)$$ (3)

**Fig 4:**
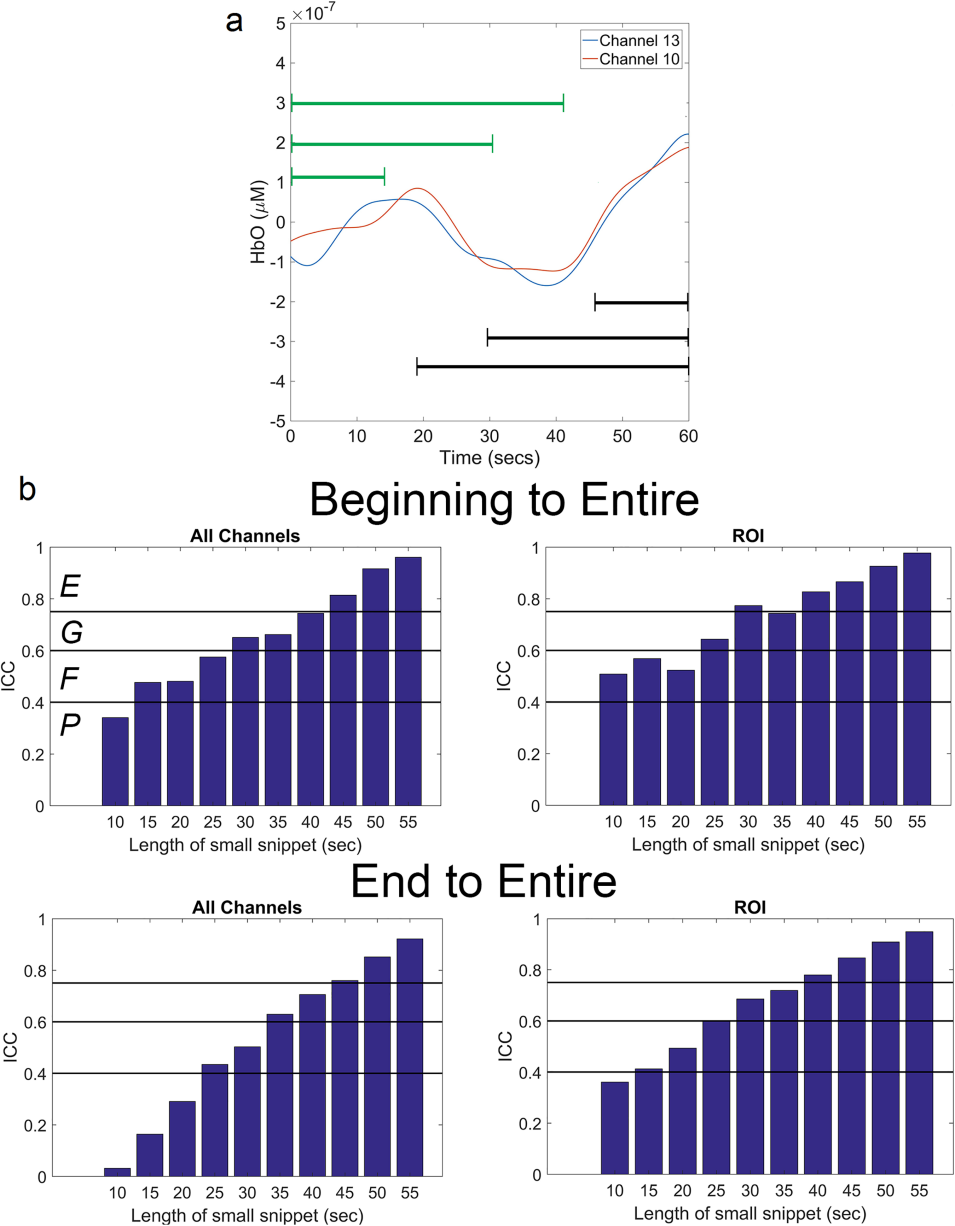
ICC analysis for reproducibility. (a) Recordings from two channels (same as Fig 3) with arrows depicting examples of the snippets used for ICC analysis. The green arrow represents snippets beginning at the start and the black arrow represents snippets terminating at the end. (b) Mean ICC results of all participants for all connectivity pairs and for connectivity pairs involving the ROI are shown. Top panel depicts results of the beginning snippets and the bottom panel depicts results for the ending snippets. Black horizontal lines indicate the thresholds for the grading scale with abbreviations in top left bar graph serving as a legend (*P* = poor, *F* = fair, *G* = good, and *E* = excellent).”

The analysis involves the correlation coefficients of the channel-pairs during the 60 seconds of silence prior to participants being presented with the auditory stimulation paradigm and the correlation coefficients during the 60 seconds of silence following the stimulation paradigm (Fig 2). While many RSFC studies employ longer recording sessions, our current research design employed a lengthy stimulation paradigm that warranted shorter recording times for resting periods to obviate problems with participant compliance, including increased motion artifact. We recorded data longer than one minute during each of the resting periods flanking the stimulation but analyzed only the minute just prior to the first stimulus and one minute following the last stimulus period to ensure stability and consistency in the testing conditions across all participants. Medvedev (2015) split the two-minute recordings in that study by instructing participants to close their eyes half-way through the session [40]. As such, we deemed one minute to be sufficient to capture the low frequency oscillations yielding RSFC with fNIRS. To ensure the consistency of the correlations, we performed intraclass correlation (ICC) analysis of the pre-stimulation condition. ICC uses analysis of variance to quantify the reproducibility of data via a ratio of between-subject and within-subject variability by comparing two data points for each participant. Correlation coefficients were calculated for snippets of varying lengths between 10 and 55 seconds starting at the beginning of the entire minute. We used these correlations as a second observation for each participant. The ICC was computed between each of those snippets and the entire minute for all connectivity pairs and for connections to the ROI. The same analysis was performed for snippets that all terminated at the end of the minute (see Fig 4). ICCs across all connectivity pairs were then averaged. ICC values between 0.4–0.59 are deemed fair, between 0.60–0.74 are deemed good, and between 0.75–1.00 are deemed excellent [41]. Even at short time lengths, our reliability is graded as fair or good. This analysis provides confidence that the correlation of brain activity among the measured brain regions is consistent throughout the measured time and would have high reproducibility with longer recording times as well. Additionally, high concordance between HbO and HbR is further evidence to the high reliability of the data.

To determine changes from pre- to post-stimulation, all values underwent Fisher transformations. Then, pre-stimulation values were subtracted from post-stimulation and the results underwent reverse Fisher transformations. From here onward when referring to correlations between channels, we will use the terms connections or connectivity.

## Results

All participants included in the study had normal/near-normal hearing. For the control group, the WRS was 99% and the SRT was 15dB HL. For the tinnitus group, the WRS was 100% and the SRT was 19dBHL. Independent t-tests revealed no differences in audiologic data between the two groups (p = 0.10). Pearson correlation coefficients were also performed between hemodynamic responses and age, hearing threshold and audiogram findings during the stimulation paradigm conditions (750Hz, 8000Hz, BBN, silence). There were no significant correlations found in either ROI or “n-1” non-ROI. Furthermore, all participants were determined to have adequate fNIRS signals and consistent headband placement from pre- and post-experimental photographs. To assess the effect of age on our data, we performed linear regression analysis of age against mean connectivity for all ROI and “n-1” non-ROI for pre- and post-stimulation and found no statistically significant correlations. There was no statistical significance between the ratio of genders (p = 0.96). Linear regression was performed to asses for asymmetry between the left and right hemisphere involving ROI and “n-1” non-ROI channels for all experimental conditions and none were statistically significant. For the subsequent results, ROI and “n-1” non-ROI values refer to the pooled values of all channels corresponding to those regions, unless otherwise noted.

### RSFC in pooled ROI and pooled “n-1” non-ROI seeds

**Fig 5:**
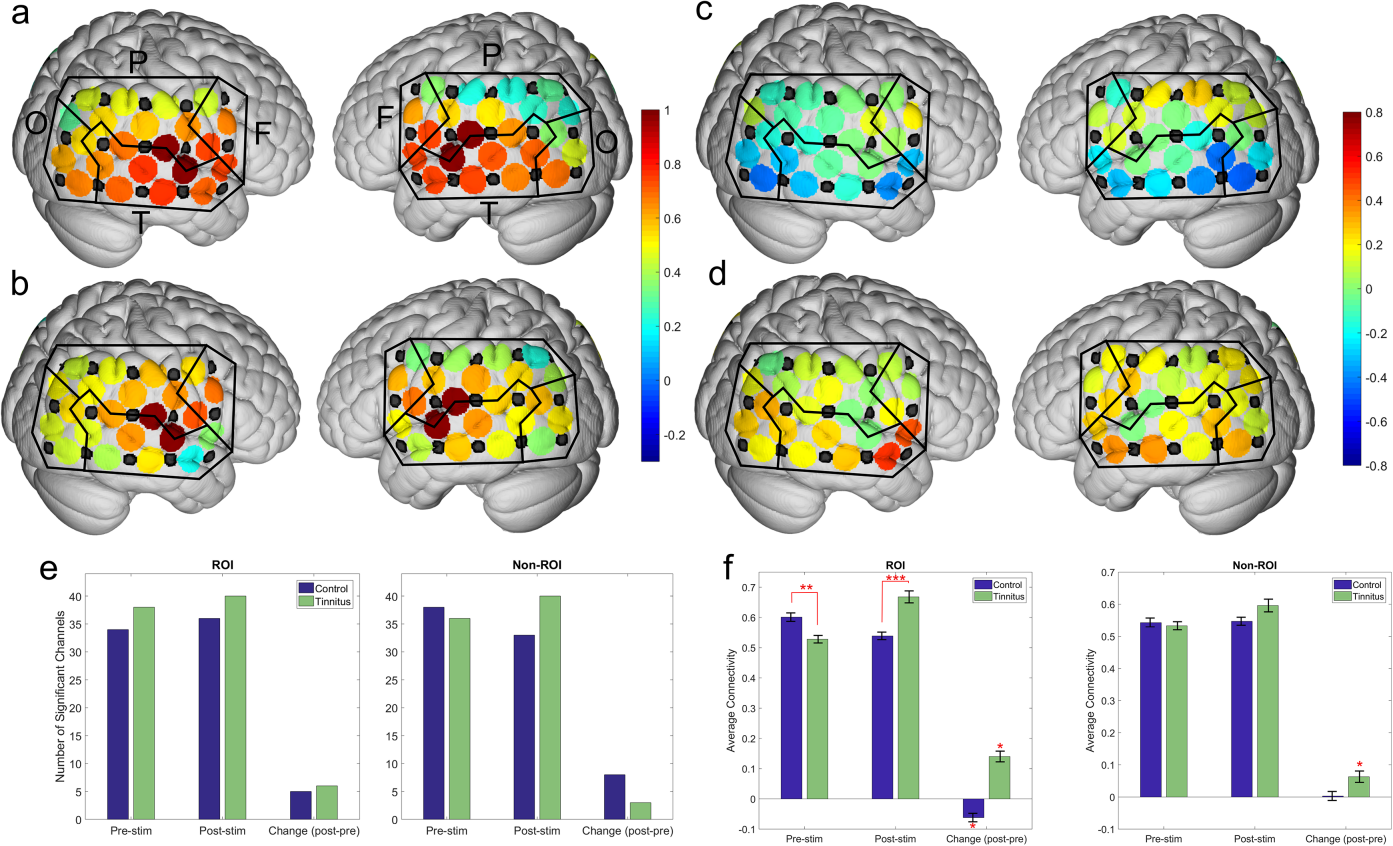
Pre-stimulation and change in ROI connectivity. Mean connections of each channel to the four pooled ROI. Black lines separate the channels into lobes. Labels in (a) serve as a legend (F = frontal, T = temporal, P = parietal, O = occipital). The mean values in the pre-stimulation condition for (a) controls and (b) tinnitus. The mean change from pre- to post-stimulation in (c) controls and (d) tinnitus. Number of statistically significant connections (e) and average connectivity (f) to the ROI and to the “n-1” non-ROI for the depicted conditions for both control and tinnitus participants. In the pre-stimulation conditions ((a) and (b)), each hemisphere contains two values equivalent to one at the locations of the ROI since the connectivity of a channel to itself results in a connection of one. In the change plots ((c) and (d)), the areas corresponding to the ROI show a connectivity of 0. Asterisks denote significance level, one (*): p<0.05; two (**): p<0.01; three (***): p<0.001.

The following analysis was performed using the ROI and the “n-1” non-ROI as seed regions. As a reminder, since ROI and “n-1” non-ROI values were pooled across 4 channels, each pooled seed region has 40 correlation data points instead of the 43. For the remainder of the manuscript, when referring to the pooled values of the four ROI seeds we will use the terms ROI seeds or ROI values (and the same corresponding nomenclature for “n-1” non-ROI). Prior to stimulation, the average connectivity of the pooled ROI in controls was higher than in tinnitus participants (0.601 vs. 0.528; p<0.005). Following stimulation, the average connectivity of the ROI was lower in controls than in tinnitus participants (0.539 vs. 0.668; p<0.0001). The increase in average connectivity of tinnitus participants and the decrease in controls were also significant (p<0.01, p<0.0001). There were no differences between tinnitus and controls in the number of channels showing statistically significant connectivity to the ROI during either condition. Interestingly, all channels that changed significantly in tinnitus exhibited an increase in connectivity, while all channels that changed in controls exhibited a decrease in connectivity. There was no difference in the average connectivity of the “n-1” non-ROI between tinnitus and controls during either condition. The increase in connectivity in “n-1” non-ROI of tinnitus was significant (Fig 5E and 5F).

**Fig 6:**
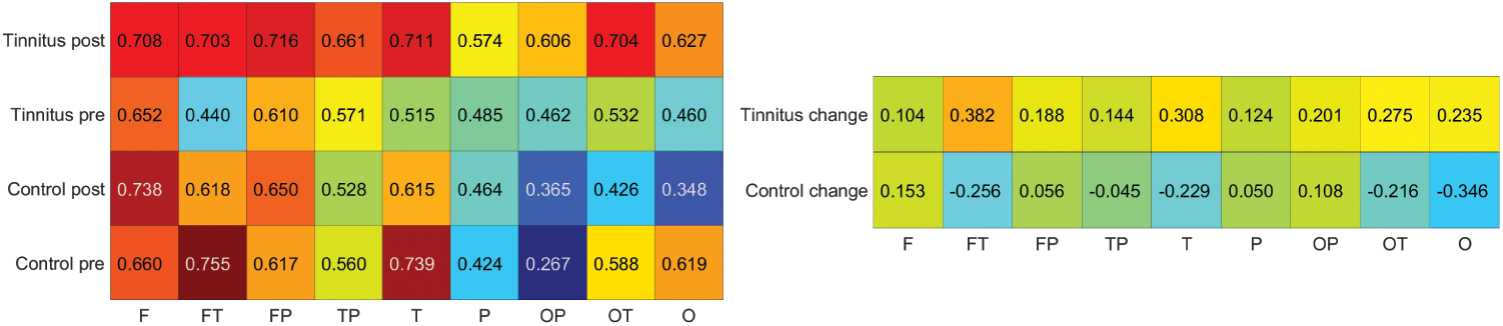
Average connectivity of ROI to the various cortical regions under multiple conditions. Y-axis indicates which type of participant and which condition (change refers to post-stimulation minus pre-stimulation). The X-axis indicates the measured region. Warmer colors in the left panel denote high connectivity, and in the right panel they denote increases in connectivity. Cooler colors in the left panel denote low connectivity and in the right panel they denote decreases in connectivity. To create the right panel, the numbers in the left panel underwent Fisher Transformation, were subsequently subtracted, and then transformed back to Pearson correlation coefficients. F = frontal, FT = fronto-temporal, FP = fronto-parietal, TP = temporo-parietal, T = temporal, P = parietal, OP = occipito-parietal, OT = occipito-temporal O = occipital.

The pattern of connectivity of the ROI to specific brain regions varied between tinnitus and controls (Fig 5A–5D). In the pre-stimulation condition, the strength of connectivity of ROI with fronto-temporal and temporal regions was greater in controls than in tinnitus. After sound stimulation, connectivity of the ROI to the temporal, occipito-temporal, and occipital regions was greater in tinnitus than in controls. Not surprising based on the above, connectivity in tinnitus and control participants was affected by sound stimulation in different ways. All statistically significant changes seen in tinnitus were increases in connectivity, whereas all the change seen in controls were decreases in connectivity. The changes observed in tinnitus participants occurred in the fronto-temporal, fronto-parietal, temporal, occipito-temporal, and occipital regions. Conversely, changes observed in controls occurred in the temporal and occipital regions (Fig 6, Table 2).

**Table 2:** Statistically significant differences in regional connectivity.

Tinnitus pre to Tinnitus post		Control pre to Control post		Tinnitus pre to Control pre		Tinnitus post to Control post		Tinnitus change to Control change	
FT	p = 0.0125	T	p = 0.0089	FT	p = 0.0028	T	p = 0.0410	FT	p = 0.0001
FP	p = 0.0385	O	p = 0.0435	T	p < 0.0001	OT	p = 0.0004	T	p < 0.0001
T	p < 0.0001					O	p = 0.0018	OT	p < 0.0001
OT	p = 0.0122							O	p < 0.0001
O	p = 0.0336								

Comparisons of connectivity of ROI to the various brain regions. All regions with statistical significance are presented along with the p-values of the corresponding comparisons. F = frontal, FT = fronto-temporal, FP = fronto-parietal, TP = temporo-parietal, T = temporal, P = parietal, OP = occipito-parietal, OT = occipito-temporal O = occipital.

### RSFC in tinnitus and controls using all seeds

The following analysis was performed using every channel as a seed in order to investigate changes taking place in cortical regions outside of the ROI and “n-1” non-ROI as shown above. For example, instead of looking at the connection between frontal lobe and ROI, we are looking at connections from frontal lobe to all other regions. Changes in connectivity from pre- to post-stimulation were determined for every channel-pair. Then, changes with a magnitude one standard deviation greater than the mean were identified to determine where and how auditory stimulation impacts RSFC the greatest. From a total of 946 channel-pairs in each subject group, the tinnitus subgroup contained 162 channel-pairs and the control subgroup contained 170. These connections were subsequently grouped into the corresponding brain regions using the anatomical information contained in Table 1.

**Fig 7:**
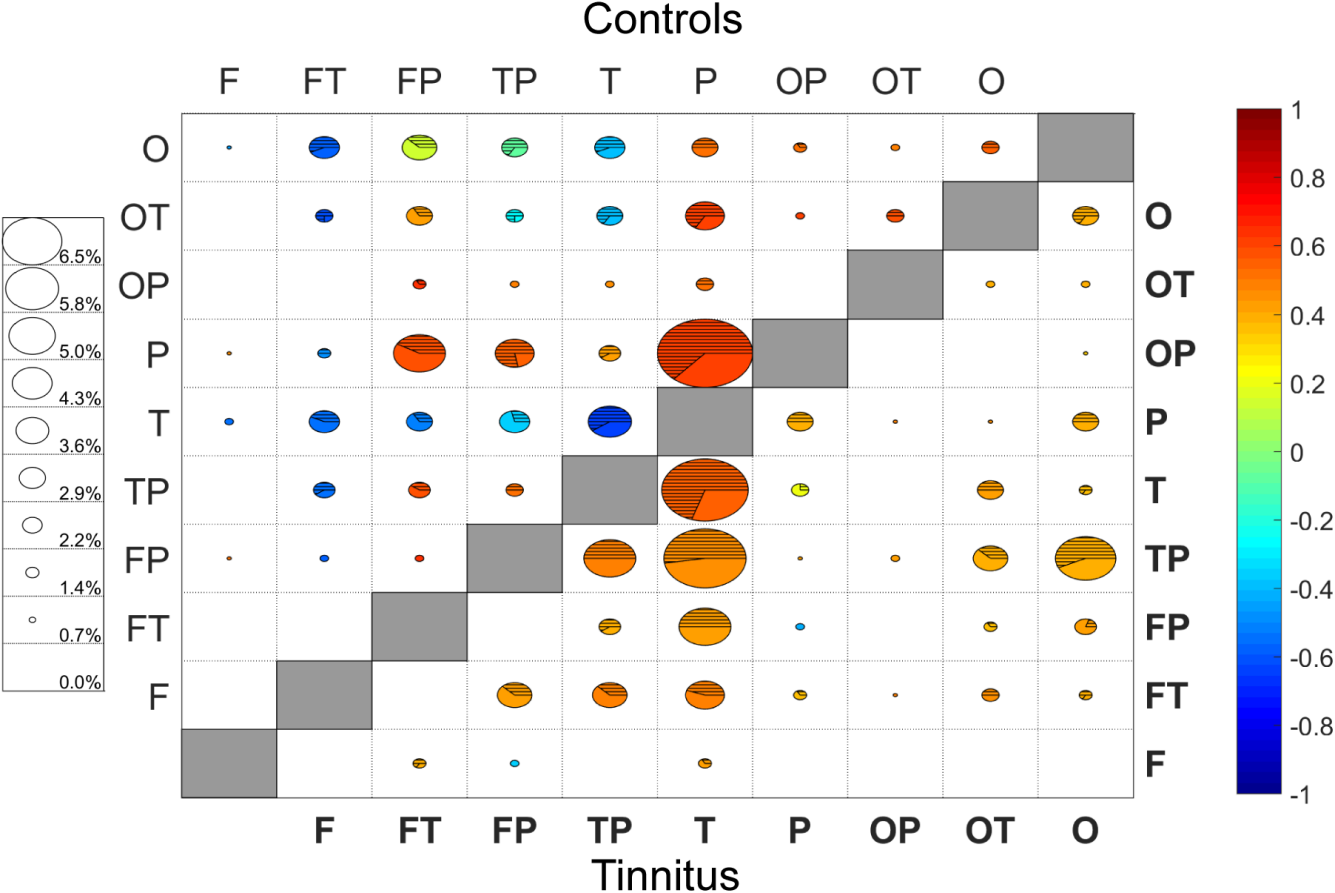
Change from pre- to post-stimulation of connections involving all measured channels. The top-left of the plot represents controls and the bottom right represents tinnitus, with the shaded boxes in middle diagonal separating the two groups. Size of each circle represents the percentage of connections that involved the two matching regions (legend on the left side of the plot for reference). The color of the circle indicates the average change of those connections (warmer colors are increased connectivity; cooler colors are decreased connectivity). The hashed region of the circle indicates the proportion of those connections that involved regions on opposite hemispheres (bilateral connections; unshaded area is unilateral only). Connections shown are one standard deviation above the mean in absolute magnitude (n = 170 in controls, n = 162 in tinnitus).

For each connection, the two cortical regions involved were evaluated to determine if they were on ipsilateral or contra-lateral hemispheres and to determine the percentage of these connections that involved each brain region (Fig 7; Table 3). There were no regions in either tinnitus or control that had a disproportionate amount of bilateral connections (statistically different from 50%), nor were there any differences between tinnitus and controls. Regarding total percentage of connections involving any particular region, the parietal region of tinnitus participants is the only one in either group that demonstrated a significant deviation from expectations (7.4% vs 18.2%, p = 0.032). However, multiple regions were disproportionally represented when comparing across groups (i.e. controls vs. tinnitus). For example, 23.46% of the connections involved the temporal region within the tinnitus group, while only 15.29% involved the temporal region of controls. This indicates that the temporal region accounted for much a larger proportion of the change seen in tinnitus participants than it did for the change seen in controls. In tinnitus, a greater proportion of change involved the temporal-parietal (p<0.01) and temporal (p<0.01) regions than in controls. Conversely, controls experienced a larger proportion of change to the parietal (p<0.001) and occipito-parietal (p<0.05) regions than in tinnitus.

**Table 3:** Connectivity between tinnitus and controls across specific cortical brain regions.

	Tinnitus			Control		
Regions	Percent	Connectivity	B/L	Percent	Connectivity	B/L
**Frontal**	2.47%	0.051	37.5%	1.47%	-0.007	80.0%
**Fronto-temporal**	12.04%	0.398**	46.2%	8.24%	-0.398**	61.7%
**Fronto-parietal**	11.42%	0.146	37.8%	13.24%	**0.300**	42.2%
**Temporo-parietal**	21.30%**	0.389**	49.3%	12.35%**	0.114**	57.1%
**Temporal**	23.46%**	0.376**	54.0%	15.29%**	-0.311**	50.0%
**Parietal**	7.40%**	**0.268**	50.0%	20.88%**	**0.437**	62.0%
**Occipito-parietal**	1.54%**	0.203	80.0%	4.71%**	**0.385**	43.8%
**Occipito-temporal**	8.03%	**0.328**	50.0%	10.88%	**0.177**	59.5%
**Occipital**	12.35%	0.348**	57.5%	12.94%	0.086**	53.3%

Summary of the connectivity between channel-pairs that showed a magnitude greater than one standard deviation (SD) from the mean in tinnitus (n = 162) and controls (n = 170). Percentage, average connectivity change, and percent of connections that cross midline. Bold indicates that percentage differs from expected or connectivity is statistically significantly different from 0, correspondingly. Double asterisks (**) indicate that the value is statistically different from the corresponding value for the other subject subgroup. No region had bilateral connections that differed statistically from 50%, and there was also no difference between controls and tinnitus in the percent of connections that were bilateral for any of the regions.

We also calculated the average connectivity change in these brain regions. There were differences between tinnitus and controls in the average change in connectivity in fronto-temporal (p<0.001), temporo-parietal (p<0.01), temporal (p<0.001) and occipital (p<0.01) regions. The largest difference was found in the fronto-temporal region, where connectivity in tinnitus increased by an average of 0.398, while it decreased in controls by an average of 0.398. For all the brain regions in tinnitus, the changes observed involved increases in connectivity, whereas multiple regions in controls experienced a decrease in connectivity.

## Discussion

In this study we hypothesized that RSFC is altered in phantom sound perception and may, in part, contribute to underlying central pathology as well as serve as a potential objective tinnitus neural correlate in humans. Using innovative fNIRS technology we observed that human participants with tinnitus display an altered pattern of spontaneous brain connectivity (RSFC) that is differentially regulated following sound stimulation as compared to non-tinnitus controls. Specifically, RSFC in tinnitus was overall lower than controls preceding the auditory paradigm, but was significantly broadened and increased in both auditory and non-auditory brain regions post-sound stimulation. This re-tuning or re-structuring of brain connectivity in human tinnitus may reflect underlying central auditory and non-auditory plasticity leading to aberrant sound processing that may contribute to phantom perception. Measured changes in magnitude and characteristic patterns of RSFC in humans using fNIRS may serve as an objective tinnitus neural correlate to ideally map brain circuits in this subjective disease and may also be used to track therapeutic intervention.

As an emerging technology, analysis of brain RSFC using fNIRS is highly reproducible and consistent when compared to similar fMRI studies allowing one to interchangeably compare results and conclusions using both imaging modalities [22]. However, due to differences in study populations, including hearing levels, and recording environments, it may be difficult to obtain consistent results when analyzing resting state data of tinnitus research across modalities [42]. Nonetheless, Chen et al [42] performed a meta-analysis of resting state studies of tinnitus involving fMRI, PET, and SPECT. They identified consistent reported changes involving the medial temporal gyrus, frontal cortex, parahippocampus, insula, cerebellum, cuneus, and thalamus. As fNIRS is only able to measure superficial cortex, our discussion will focus on these areas.

Using fNIRS, RSFC has been successfully measured in language centers of the human brain [43] demonstrating the broad application of this technology to map intact brain circuits. As a concept of brain function, changes in RSFC may also reflect neuroplasticity that is partly responsible for both adaptive and maladaptive central neural conditions [12, 13]. For example, McKay et al. [44] demonstrated that cochlear implant (CI) users with poor speech understanding have less inter-hemispheric connectivity than CI users with optimal performance and resultant normal hearing. This demonstrates that changes in RSFC may be representative of neuroplasticity affecting auditory processing. Although previous studies have investigated hemodynamic activity in human tinnitus using fNIRS [8,10], our current results are the first to examine RSFC. To date, fMRI studies have shown network dysregulation of many central pathways, including non-auditory and auditory-sensory cortices in humans with tinnitus [45]. Since phantom sound perception may originate from changes in multiple synchronized brain networks [15] that may extend beyond dedicated central auditory pathways, we investigated RSFC within various human auditory and non-auditory cortical areas (frontal, parietal, temporal, occipital fronto-temporal, fronto-parietal, temporo-parietal, occipito-parietal, occipito-temporal) under intact (non-tinnitus) and aberrant (tinnitus) conditions.

Here we observed increased RSFC between ROI and multiple non-auditory cortices in tinnitus following sound stimulation. Connectivity was decreased in controls following sound stimulation suggesting that a fundamental change in the tinnitus brain involves expanding ROI network properties to regions not dedicated to auditory processing. This finding is in line with previously reported fMRI data implicating non-auditory areas, such as limbic, prefrontal areas, nucleus accumbens and associated paralimbic structures, in the pathophysiology of tinnitus [46–49]. These brain regions may play a role in networks involved in the perception of and those involved in the emotional reaction to tinnitus. Indeed, increased gamma band in corticothalamic and corticolimbic networks may underlie the tinnitus percept [50]. Since our results show increased connectivity of non-auditory areas to the auditory cortex, they may also be representative of multi-sensory integration. This “recruitment” of non-parallel, multi-modal sensory systems has been increasingly studied in animals and humans. Multi-sensory integration is well characterized in tinnitus animal models whereby damaged auditory circuits are controlled by non-auditory sensory systems [51]. For example, auditory cortical regions are increasingly responsive to multiple sensory systems including auditory, visual and somatosensory [51, 52]. This suggests not only adaptation to overcome sensory deprivation but also implicate multiple non-auditory systems in the etiology of phantom perception. Using noise-induced animal models of tinnitus, we demonstrated that spontaneous and tone-evoked neural firing rates in primary auditory cortex are relatively unresponsive to unimodal sound stimulation alone. These neurons, however, increase or decrease their firing rates following bimodal (auditory-somatosensory) stimulation depending upon the timing and pairing order of the sensory stimulus [53]. In addition, repeated pairing of pure tones with nucleus basalis (cholinergic), locus coeruleus (norepinephrine), or ventral tegmental (dopamine) stimulation increases neural responses to the same pure tone in auditory cortex in animals [54–58]. Evidence of cross-modal plasticity within auditory and non-auditory networks has also been observed in humans using various stimuli. For example, fNIRS was recently used to demonstrate that profoundly-deafened humans have higher cross-modal plasticity in temporal lobe to visual stimuli than normal-hearing adults [59]. Moreover, trans-cranial direct current stimulation produces changes in RSFC of primary auditory cortex in tinnitus but not in control participants [60]. Finally, it should be noted that cross-modal plasticity may be dependent on the synchrony or timing of the stimuli presented. Wiggins et al. [61] used fNIRS to demonstrate that synchronous audiovisual stimuli produced decreased activation in visual cortex as compared to asynchronous or unimodal visual stimulation. This suggests that specific timing properties of multimodal stimuli should be investigated in future studies.

Here we observed pre-auditory decreases in RSFC between ROI and fronto-temporal and temporal cortices and a downward trend in connectivity with the occipital lobe. This is congruent with fMRI results demonstrating poor connectivity between auditory cortex and occipital lobe [62] and decreased connectivity in right auditory cortex, left frontal and bilateral occipital regions [49]. Decreased connectivity between these regions during silence suggests that those specific networks may not play a large role in phantom perception during “static” auditory periods. Conversely, during “dynamic” periods with or after sound stimulation, these multi-sensory networks may contribute to phantom perception; a concept supported in our results by increased connectivity between the ROI and the frontal, temporal and occipital lobes after sound in tinnitus. These findings corroborate the increased resting state neural activity and abnormalities in functional connectivity that are consistently seen in the frontal and temporal lobe of tinnitus brains [42]. The reciprocal decrease in RSFC in controls, particularly with the occipital lobes, under the same stimulated conditions implicates the role of the visual system in the pathophysiology of the disease. Since spontaneous activity reflects experience and contextual influences and can affect local processing and perception [63, 64], increased connectivity of the ROI with multiple brain areas suggests that non-auditory cortices play an increased role in sound processing in tinnitus.

Our data also support the notion that non-auditory regions contribute to chronic tinnitus perception through large-scale networks [45, 65] and changes in connectivity. Five out of nine non-ROI cortical regions measured after sound stimulation in tinnitus exhibited increases in connectivity to the rest of the regions measured. The fronto-temporal and temporal regions exhibited the largest differences in change following stimulation as RSFC in both regions increased in tinnitus and decreased in controls. Large decreases in the control temporal region was seen following sound stimulation suggesting temporal and fronto-temporal regions as key contributors to altered central networks. While it is currently unclear whether altered RSFC in these particular regions plays a role in phantom percept etiology, Chen et al. [66] have shown that aberrant connectivity involving the non-auditory superior frontal gyrus (SFG) with auditory and other non-auditory cortices in tinnitus may be a key locus to pathology. Furthermore, activation of the right middle temporal gyrus has been shown to be increased during tinnitus perception but not during masking conditions using PET [67].

Contrary to previous studies, our data found no difference in RSFC between the hemispheres [45, 65, 66, 68, 69]. This is most likely due to the laterality differences found in those studies often involving deeper brain regions not readily accessible in this study due to fNIRS depth of penetration that is limited to outer cortical regions.

## Limitations

A significant limitation of fNIRS lies in its inability to record hemodynamic activity from brain regions deeper than the outer cerebral cortex. Important connectivity differences seen in tinnitus may involve sub-cortical areas such as the limbic system [70], therefore, future fNIRS studies will require adaptation of this innovative technology to expand brain surveillance. Another limitation of our study was the shorter pre- and post-stimulation baselines of one minute each. While many studies investigating RSFC require longer average recording times, our current research design that employed a lengthy interval stimulation paradigm warranted shorter baseline recording times to obviate problems with participant compliance including increased motion artifact. ICC analysis comparing the full minute to snippets within the minute indicated fair reproducibility with snippets as short as 20 seconds. This indicates that the data is consistent throughout the entire recording and provides confidence that it would provide the same results at higher recording durations. Furthermore, statistical analysis of the recordings demonstrated high concordance between HbO and HbR and a high level of consistency of the data even at shorter recording lengths. Future studies will be designed to implement longer baseline recordings to better characterize the temporal nature of change in RSFC under experimental and control conditions. Lastly, there was a discrepancy between the ages of the two groups. Nonetheless, since Pearson regression analysis revealed no statistically significant correlations between age and hearing levels or mean ROI connectivity we feel confident that the results represent changes associated with tinnitus.

## Conclusion

Networks uniquely identified in tinnitus suggest that aberrant patterns of RSFC involving auditory and non-auditory regions may be essential to the central pathophysiology [45]. As such, RSFC may also serve as an important potential objective neural correlate of tinnitus in humans. Identifying a reliable, objective measure in humans with this subjective pathology could improve diagnosis, prognosis and treatment monitoring [68]. fNIRS is uniquely qualified to serve this role due to its portability and low cost allowing it to be readily available in clinics and to be a viable option in low-resourced countries. Furthermore, fNIRS has higher spatial resolution than EEG and higher temporal resolution than fMRI, while eliminating important confounders in the study of tinnitus since it operates virtually silently [5].

To our knowledge this is the first study to examine RSFC in human tinnitus using fNIRS technology. Our data demonstrate significant changes in RSFC involving both auditory and non-auditory cortical regions in human tinnitus and controls. Altered RSFC observed throughout the brain following sound stimulation in tinnitus suggests that multiple central auditory and non-auditory regions may contribute to phantom perception. Future studies using fNIRS will closely investigate how and where multisensory processing may influence RSFC, a reliably measurable marker that may also serve as an objective correlate of human tinnitus.
